# Activating faith: pro-environmental responses to a Christian text on sustainability

**DOI:** 10.1007/s11625-022-01197-w

**Published:** 2022-08-22

**Authors:** Christopher D. Ives, Clark Buys, Charles Ogunbode, Matilda Palmer, Aneira Rose, Ruth Valerio

**Affiliations:** 1 School of Geography, University of Nottingham, University Park, Nottingham, NG7 2RD UK; 2 Tearfund, 100 Church Rd, Teddington, TW11 8QE UK; 3 School of Psychology, University of Nottingham, University Park, Nottingham, NG7 2RD UK

**Keywords:** Religion, Values, Pro-environmental behaviour, Sustainability, New Ecological Paradigm, Worldview

## Abstract

With growing attention on the importance of values, beliefs and worldviews in shaping environmental outcomes, there remains little research on religion and sustainability transformations. We explored the impact of the Archbishop of Canterbury’s environmentally themed Lent Book 2020 “Saying Yes to Life” on environmental values, attitudes and behaviours of lay Christians. An online survey administered before and after reading the book assessed environmental values, New Ecological Paradigm (NEP), connectedness to nature and environmental behaviours, and collected open responses to questions about participants’ perceptions. Follow-up focus groups were also held to understand experiences of cognitive and behavioural change. Analysis of paired data revealed significant increases in environmental behavioural intentions after completing the book, especially for energy use, food and recycling. Some evidence for strengthening of NEP scores and connectedness to nature was also found. Open text responses corroborated with quantitative measures of behaviour change. Additionally, the majority of participants reported some form of reinforcement, confirmation, or further development or change in their beliefs and attitudes. This included a reduction in anthropocentric beliefs and greater appreciation of and obligation towards the natural world. Focus group discussions revealed diverse participant experiences, including having pre-existing theological beliefs affirmed, responding with new practical actions, connecting with spiritual experiences, and discovering systemic origins of unsustainability. Findings suggest potential for environmental interventions within religious contexts to shape mindsets, integrate theological views with environmental concerns, activate latent beliefs, and initiate and sustain pro-environmental behaviour. More intentional engagement with religion may facilitate transformative change for sustainability internally and externally, and across individual, organisational and societal domains.

**Supplementary Information:**

The online version contains supplementary material available at 10.1007/s11625-022-01197-w.

## Introduction

Sustainability scholarship has begun to highlight the deeply ingrained values, beliefs and worldviews that underlie the twin crises of biodiversity loss and global warming. These span scales from societal economic paradigms that emphasise material consumption and productivity, to individual anthropocentric beliefs and apathetic or exploitative attitudes towards nature. This has led some scholars to identify systems of customs, values and social norms as lying at the root of planetary breakdown (Otto et al. 2020). Others have highlighted the importance of morality—often with roots in religious frameworks and worldviews—in shaping environmental decisions and actions, including among groups that may not be overtly environmentally orientated (Lau et al. 2021). One feature of human society that has a particularly prominent role in shaping systems of values, beliefs, worldviews and behaviour is religion (Ives and Kidwell 2019). With an estimated 85% (Pew Research Centre 2017) of the global population adhering to religious faith of some kind, religion has immense potential to shape cultural values, norms and practices within human societies.

The rate, scale and significance of environmental degradation across the planet has given rise to the “transformative agenda”: a recognition that responses to these challenges require wholesale, deliberate, system-wide change rather than incremental improvements in the status quo (O’Brien 2012; Scoones et al. 2020). Language of transformation now pervades sustainability literature, in part, due to its foregrounding in the UN Agenda 2030, which introduced the Sustainable Development Goals. While most research on sustainability transformations has focussed on external change, a small but growing literature is considering the relevance of “inner transformations” (or inner transitions) (Wamsler et al. 2021). Proponents of this perspective argue that transformations to sustainability must be accompanied by concomitant change in our “inner worlds” (Ives et al. 2020)—people's values, beliefs, attitudes and worldviews. Values have been proposed as “deep leverage points” whereby shifts in values can lead to and support broader changes in system structures and behaviours (Horcea-Milcu et al. 2019). Indeed, the IPBES Global Assessment has identified priority “leverage points” for enabling societal transformation for sustainability as including embracing “diverse visions of a good life” and “unleash[ing] latent values of responsibility to enable widespread action” (Chan et al. 2020, p. 7). There is a need, therefore, for further research into what such values and visions are, and how they are formed, operationalised and potentially changed in particular social settings and contexts.

An extensive body of literature has explored the varied moral and ethical positions of religious traditions on environmental matters (e.g. Grim and Tucker 2014; Jenkins et al. 2016). However, there is a need for more research on how people *actually* behave rather than the ethical beliefs that inform how people *ought* to behave (Berkes 2011). Empirical studies that have sought to do this in the context of religion and environmental behaviour have revealed a complicated and oftentimes ambivalent relationship. For example, Hayes and Marangudakis (2000) found that Christians and non-Christians did not differ in their concern for the environment and that religious identification was inconsistent in predicting environmental behaviour among participants from four Western developed countries. In the North American context—where a substantial amount of research on this topic has been conducted—Christians typically report lower levels of pro-environmental behaviours (Clements et al. 2014). However, this pattern varies according to religious affiliation (Arbuckle 2016), and is largely a product of a complex interaction between political ideology and religiosity. Indeed, while there is an association between conservative Protestant Christianity and right-wing politics in the USA (Zaleha and Szasz 2014), a recent study has shown that increased levels of religiosity can counteract the negative influence of political conservatism (Peifer et al. 2016). Complex associations between religion, politics and historical movements are evident elsewhere around the world, for example, the reluctance of many Muslim religious leaders in Pakistan to embrace environmentalism because of its perception as a “Western” agenda (Rizvi 2005). Overall, a recent meta-analysis of studies of relationships between religion and “sustainable consumption” behaviours between 1998 and 2019 concluded that much of the mixed and contradictory relationships observed are due to methodological differences (e.g. defining and operationalising religion in different ways), and that many constructs moderate religion-behaviour relationships, such as dominion or stewardship beliefs, political orientation, or life satisfaction (Orellano et al. 2020). Indeed, with careful control of potential moderating and confounding factors, a recent global analysis identified an overall positive relationship between religiosity and pro-environmental behaviour across cultures and geographical settings (Zemo and Nigus 2021).

In recent years, there has been a proliferation of faith-based initiatives that have sought to expound the green virtues of various faith traditions, encourage religious followers to adopt pro-environmental lifestyles, and engage seriously with global environmental discourse and policy. Examples include publications such as the Papal Encyclical Laudato Si’ (On Care for our Common Home) (Pope Francis 2015), Thich Nhat Hanh's publication “Zen and the Art of Saving the Planet” (Hanh 2021), the 1986 Assisi Declarations (messages on humanity and nature from Buddhism, Christianity, Hinduism, Islam and Judaism) (Alliance of World Religions 1986) and the later 2015 Bristol Faith Commitments representing 24 religious traditions (Alliance of Religions and Conservation 2015), along with the UNEP Faith for Earth’s “Call to Action” (United Nations Environment Programme 2020). These and similar initiatives have led some scholars to suggest a global “greening of religion” is underway (Chaplin 2016). Other scholars, however, have been more sceptical, suggesting that empirically, most of this “greening” has been isolated to institutional rhetoric and action by a small community of highly visible and vocal actors that do not represent the values and behaviours of the majority of religious followers (Taylor et al. 2016; Taylor 2019).

Irrespective of whether world religions are responding sufficiently to the present global environmental crisis, there is growing consensus that communities of faith must respond and that they have unique and powerful contributions to offer. Indeed, the UN climate chief, Christiana Figueres said that “It is time for faith groups and religious institutions to find their voice and set their moral compass on one of the great humanitarian issues of our time” (Figueres 2014). Scholars have pointed to the potential of religion to enable global environmental stewardship because of the cultural, institutional, and spiritual resources they offer (Hitzhusen and Tucker 2013; Mcleod and Palmer 2015). Indeed, the recent IPCC Working Group III report asserted that “religion could play an important role in enabling collective action on climate mitigation” (IPCC 2022). As such, there have been calls to more effectively connect climate science with religious worldviews (Muller 2021). Psychologically, religious perspectives shape people’s beliefs about human–nature relationships, including what is understood as sacred and right (Sachdeva 2016). Religious traditions also have potential to promote stewardship beliefs, recognise nature as sacred, and attach ultimate and eternal significance to environmental care (Preston and Baimel 2021). From a socio-technical systems standpoint, religious institutions can be understood as enabling environmental action in three domains: public campaigning (driving structural change), materialising outcomes (“greening” institutional activities), and disseminating values (promoting particular values and worldviews to religious followers and wider society) (Koehrsen 2018). Yet, these pathways are largely theoretical, and there is scant research on how interventions can be designed and implemented to activate such changes.

As reviewed above, most prior empirical research on religion and the environment has been observational, typically identifying associations between strength or type of religious belief with environmental behaviour. In contrast, there has been little exploration of how attitude and behaviour change might happen *within* religious contexts. In the present study, we explored whether and how an intervention that presented messages in the context of the Christian faith can enable shifts in pro-environmental attitudes or behaviours among believers. Most environmental messaging to date has been techno-scientific—focussing on scientific explanations of environmental breakdown and required technological solutions—as well as secular—ignoring religious values, beliefs and worldviews that members of the public hold. We were interested in whether presenting environmental messages through the lens of religious faith could offer a new pathway for shaping mindsets and behaviours, through engaging individuals holistically, and activating non-utilitarian justifications for pro-environmental action such as care, compassion, reverence, moral responsibility, and love.

We pursued the following research questions. First, does engaging with religiously framed literature on environmental sustainability by people of faith precipitate changes in environmental behaviours, and if so, are changes in some behavioural domains more pronounced than others? Second, are changes in beliefs, values, and attitudes observed? Finally, are internal phenomena (beliefs, values, worldviews) associated with external behaviour change? These questions were pursued via a mixed method programme of research. Results are used to propose a model of how environmentally relevant beliefs and cognitions may change in religious contexts. The article concludes with a discussion of how religious communities can be effectively engaged for action on sustainability.

## Methods

### Description of intervention (“Saying Yes to Life”)

The intervention for this study was the book titled “Saying Yes to Life” (SYTL), authored by Dr Ruth Valerio, and published as the Archbishop of Canterbury’s Lent book 2020 (Valerio 2020). It was read by Christians in the UK (and around the world), and engaged with in different ways (discussion groups, church teaching, etc.). Supplementary online materials were also provided (https://spckpublishing.co.uk/saying-yes-to-life). The text was structured around the creation story in the Christian Bible, and covered environmental challenges such as water scarcity, air pollution, land degradation, biodiversity loss, and energy use. Additionally, theological themes were discussed such as dispensationalist beliefs (i.e. beliefs that the physical earth is temporary), dualism between matter and spirit, and ethics of stewardship. Stories of the experiences of communities in the Global South who are grappling with impacts from climate change and environmental degradation were also included. Each chapter ended with discussion points and a contribution for spiritual reflection (e.g. a prayer). As such, the book was designed to stimulate thought and reflection, and elicit a practical response towards sustainable behaviour in the reader.

### Survey design

The survey was designed to elicit data on responses to engaging with the book and collect descriptive information about participants (see supplementary material for the full survey instrument). One question asked how people engaged with the book, e.g. reading alone, discussing with others, or aiding personal prayer. Attitudes towards the environment were measured using the revised New Ecological Paradigm scale (NEP-R) (Dunlap et al. 2000) via a 5-point Likert scale. 29 items relating to self-reported environmental behaviour were asked, based on Melo et al. (2018) and Whitmarsh and O’Neill (2010). Participants were presented with the question “Below are some personal actions that relate to the environment. How often do you do the following?”. A modified version of this question was provided for the follow-up survey, administered at a later date (see Sect. 2.3 for details). The modified question was “After engaging with "Saying Yes to Life", how often do you perform, or intend to perform the following actions?” Items were grouped according to the following categories: habitual energy behaviours (four items), one-off energy behaviours such as switching to a green energy supplier (four items), transport behaviours (five items), shopping (four items), food behaviours (three items), environmental activism (three items), recycling (five items), and spirituality (one item). Personal values were measured via a scale based on Stern et al. (1998)—an adaption of Schwartz’s (1992) universal human values instrument. Survey items were structured according to three value orientations, namely Egoistic, Altruistic and Ecocentric values. These were measured on a 9-point Likert scale. Connection to nature was measured via the shortened Nature Relatedness scale (NR-6) (Nisbet and Zelenski 2013).

Free text responses to three questions about how reading SYTL shaped participants' perceptions were recorded. These covered (i) conceptions of God and nature, (ii) people’s thoughts on Church, mission^1^ or how Christians should live, and (iii) ideas of how society should respond to COVID-19. Information was also collected on participants’ faith context, including the importance of religion in their lives, frequency of participation in corporate and personal religious activity, measures of biblical literalism (taken from Schultz et al. 2000), and denominational affiliation. Finally, socio-demographics including age, gender, education, economic status, and political persuasion were collected. Economic status was self-reported using a 9-point Likert scale between “lower income” and “upper income”. Political persuasion was measured on a 7-point Likert scale, with respondents indicting where their “political views” stood on a spectrum between “Extremely liberal” and “Extremely conservative”. Ethics approval was granted by the School of Geography, University of Nottingham in March 2020.

### Survey administration

Surveys were administered online using Qualtrics. They were advertised using social media, namely Twitter and Facebook—the latter via a group set up by the book’s author as an online forum for discussion and interaction. A pre-test, post-test design was employed whereby participants were asked to respond both before reading the book (T1), and after completing it (T2). The survey was modified accordingly so that the first iteration included a subset of the full complement of questions for key baseline data to be collected. The first baseline (T1) survey was open between 6 March and 2 April 2020, and the follow-up (T2) survey between 21 April and 5 June 2020. The total number of responses was 245: 81 before and after (pre- and post-test), 112 before only (pre-test), and 52 after only (post-test). In the UK, the survey coincided with the first wave of the COVID-19 pandemic, with government restrictions on social activity coming into force after the baseline survey had been released. Consequently, behavioural intentions were included in the phrasing of the follow-up survey question on self-reported behaviour because government rules would have impacted participants’ routines and lifestyles. While this was not ideal, research has shown that behavioural intentions moderately predict actual behaviours (Sheeran and Webb 2016). The pandemic also meant that a higher proportion of participants failed to complete the second survey than anticipated, and many settings planned for group engagement and discussion could not go ahead. To accommodate this, an additional open question was added to ask about participants’ reflections on the pandemic and how they saw its relationship to themes covered in the text.

### Participant profile

The majority of respondents were from the UK. Ages ranged from 18–24 years to 75–84 years, with a mode of 45–54 years old. There was an over-representation of female respondents (157 female, 57 male, 1 other, 30 prefer not to say/no response). The cohort was well educated, with 76% holding an undergraduate degree or higher degree. The majority of respondents identified as Anglican (59%), with the next largest groups Baptist and “other” (12% each), followed by “no denominational affiliation” (7%). The high representation of Anglican/Church of England respondents is likely due to the text primarily being promoted within Church of England settings. Nevertheless, as a religious institution, the Church of England is known to encompass diverse theological and socio-political perspectives. A range of political persuasions was recorded with a bias towards liberal/progressive views. 60% of participants self-identified as “very liberal” or “somewhat liberal”, 23% as “centre”, and 18% as “somewhat conservative” or “very conservative”. Most participants recorded themselves as middle income, with 59% rating themselves as 4, 5, or 6 on the 9-point scale.

### Focus groups

To further explore participants’ experiences of engaging with the text and understand the process of observed change, participants were given the opportunity to partake in a focus group discussion. Consenting survey respondents were invited by email, with 16 participants accepting. 15 respondents were available to participate and were organised into three groups based on availability. All groups were facilitated by the same researcher for consistency. Due to the difficulty of arranging focus groups within COVID-19 social distancing regulations, focus groups were held online via Microsoft Teams. Participants were grouped on availability as opposed to other factors (e.g. age, denomination), or the use of pre-existing networks. Discussions followed a semi-structured format, following protocols set out by Longhurst (2010). Key questions were formulated to define specific topics of discussion, namely (i) personal opinions of the text, (ii) previous thoughts on environmental issues and whether beliefs and attitudes had changed in response to the text, (iii) any changes in environmental behaviours, (iv) impact of the text on knowledge and understanding of environmental issues, and (v) whether the text was a useful tool in helping to explore personal beliefs, values, attitudes, and behaviours from a theological perspective. However, conversation was not restricted to these topics, but actively encouraged to diverge towards other specific points of interest (Wolgemuth et al. 2015) as the participants conversed with each other.

### Quantitative data analysis

To test statistically for differences in quantitative responses to survey questions after reading the book, only the 81 complete responses with records at both time points (T1 and T2) were analysed. Sensitivity analysis using G*Power (Faul et al. 2007) indicated that a sample size of 81 is adequately powered (1−*β* = 0.80, *α* = 0.05) to detect a small population effect size (Cohen’s *d*_*z*_ = 0.32). One-sided paired *t* tests (H1: T2 > T1) were used to test for the effect of the intervention (reading the text) on a number of dependent variables: environmental behaviours and behavioural intentions, New Ecological Paradigm, values, and Nature Relatedness. Additionally, due to the small sample size, Bayes factors were estimated to determine the quality of evidence underlying any observed effects (Biel and Friedrich 2018).

To account for potential moderating effects of co-variates on behavioural changes, correlations between behaviour change and socio-demographics, political persuasion, religiosity, values and Nature Relatedness were assessed. While it is unusual to consider personal values and Nature Relatedness as both dependent and independent variables, the fact that the intervention sought to engage with deeply held beliefs and worldviews meant that exploring changes in constructs further down in the cognitive hierarchy (Rokeach 1973) was justified, and would contribute to contemporary debates over the malleability of these in environmental and sustainability contexts (Ives et al. 2017; Kendal and Raymond 2019; Manfredo et al. 2021). Statistical analyses were performed using IBM SPSS Statistics 26 and JASP.

### Qualitative data analysis

Free text responses from the T2 survey were collected from 133 participants, with 52 of these only completing the second iteration of the instrument. Reflexive thematic analysis was used to identify codes and emerging themes that corresponded to particular questions. Thematic analysis was also conducted on transcribed focus group recordings using NVivo 2.1 software to identify patterns within the data (Braun and Clarke 2006). This inductive form of analysis allowed for flexible interpretation of participant responses.

## Results

### Statistical analysis

Results of tests of differences in self-reported pro-environmental behaviours, New Ecological Paradigm (NEP), Nature Relatedness (NR), and values are reported in Table 1. Tests showed significant increases in pro-environmental behaviours and behavioural intentions after reading the text. This was true across a number of behavioural domains, especially recycling, food choices, and habitual energy practices. There was some evidence of strengthening NEP and NR, but this was not as pronounced as behavioural changes. No significant differences in transcendental value orientations (biospheric, altruistic, and egoistic values) were observed.

**Table 1:** Paired *t* tests (two-tailed) and Bayes factors (one-sided H1: T2 > T1) to assess changes following engagement with the text

DV	M (SD)		*t*	*df*	Sig	*d*_z_	BF_10_	BF_10_ interpretation
	Pre-test (T1)	Post-test (T2)						
Pro-environmental behaviour (aggregate)	0.68 (0.08)	0.73 (0.07)	− 6.27	80	< 0.001	− 0.70	1.368e + 6	Very strong
Habitual energy behaviour	0.76 (0.12)	0.82 (0.12)	− 4.41	80	< 0.001	− 0.50	1154.20	Very strong
One-off energy behaviour	0.60 (0.11)	0.66 (0.17)	− 3.60	77	< 0.001	− 0.41	82.17	Very strong
Transport behaviour	0.64 (0.14)	0.66 (0.13)	− 1.23	80	0.111	− 0.14	0.447	Anecdotal
Shopping behaviour	0.76 (0.12)	0.80 (0.10)	− 3.37	80	< 0.001	− 0.37	41.64	Very strong
Food behaviour	0.69 (0.15)	0.73 (0.13)	− 4.47	80	< 0.001	− 0.50	1386.14	Very strong
Environmental activism	0.43 (0.18)	0.48 (0.15)	− 3.14	80	0.001	− 0.35	22.03	Very strong
Recycling	0.82 (0.18)	0.91 (0.13)	− 4.59	80	< 0.001	− 0.51	2110.39	Very strong
Spirituality	0.71 (0.21)	0.76 (0.19)	− 1.98	79	0.026	− 0.22	1.51	Anecdotal
NEP	3.88 (0.39)	3.96 (0.41)	− 2.01	80	0.024	− 0.22	1.62	Anecdotal
Altruistic value	5.74 (0.98)	5.65 (1.01)	− 0.81	78	0.419	− 0.09	0.27	Anecdotal
Biospheric value	5.19 (1.19)	5.34 (1.11)	1.68	78	0.097	0.19	0.05	Anecdotal
Egoistic value	1.82 (1.12)	1.81 (0.99)	− 0.06	78	0.950	− 0.01	0.13	Anecdotal
Nature-relatedness	4.00 (0.69)	4.11 (0.58)	2.48	78	0.015	0.02	0.04	Anecdotal

BF_10_ indicates weight of evidence in support of H1 in the data. Values between 0.33 and 3 are considered indicative of inconclusive or “anecdotal” evidence. *d*_z_ is reported here to maintain consistency with sensitivity power analysis

To understand associations between observed behavioural differences and socio-demographics (age, gender, income, education), political persuasion, religiosity, biospheric values and Nature Relatedness, Pearson correlation tests were performed (see Table 2). Younger people and those with conservative political views were more likely to exhibit greater change in behaviour after engaging with the text. Biospheric values and Nature Relatedness scores were inversely related with change in behaviour, suggesting a possible ceiling effect.

**Table 2:** Zero-order correlation of psychological and demographic covariates with difference in self-reported behaviours (T2 − T1)

	Association (*r*) with difference in behaviour (T2 − T1)
Gender (male)	0.02
Age	0.23*
Education	− 0.06
Household income	0.00
Politics (higher values indicate political conservatism)	0.32**
Religiosity	0.00
Altruistic value (T1)	− 0.08
Biospheric (T1)	− 0.29**
Egoistic (T1)	0.11
Nature-relatedness (T1)	− 0.36**

Cell entries are Pearson correlation estimates

*p < 0.05, **p < 0.01

### Open survey responses

#### Behavioural impacts

Participants were invited to respond to the question: “Did ‘Saying Yes to Life’ change the way you intend to live your life? If so, how?” A variety of pro-environmental lifestyle changes was evident from the open responses to the survey. These included changes to individual behaviours, and collective actions, along with reinforcing existing behaviours and shifts in concern and/or awareness of environmental issues. It is noteworthy that the vast majority of respondents expressed a concern around the need for broad societal change as a result of COVID-19, which may have coloured some of their statements to individual changes (e.g. “Society should not go back to normal. This should be the turning point.”).

Approximately 80% of respondents felt that SYTL had either reinforced or changed the way they intended to live. While it was not possible to determine the degree to which behavioural intentions were translated to lifestyle changes due to the study coinciding with COVID-19 lockdowns, qualitative responses shed light on behavioural priorities and processes of change identified by participants. Individual behaviour changes mirrored many of those covered in the closed question section of the survey, such as making more sustainable consumer choices regarding manufactured products (e.g. “Going forward, I think I will put more thought into the products I buy and where they are sourced. I'll think more about whether it is made of recycled materials”), energy (“I was prompted to switch to a renewable electricity supply after reading an early chapter”), and food (“We have also committed to only eating meat max 3 times a week and ideally getting it from a local butchers”). Interestingly, behaviours also extended to personal religious practices, with one respondent stating “I will thank God for my water. I will listen more intently to subjects on environmental issues, being now prepared to add my voice and to pray”. While not all participants reported behaviour change, there was evidence that the text may have helped to overcome cognitive barriers: “It really made me think about actually acting on the things I've been wanting to change in my life for a long time but never had the motivation to do”. Collective action was also evident. Examples spanned encouraging pro-environmental initiatives within their church community (“Would like to read & discuss the book with others in my church and bring about change in our church. Switch to green power, be more aware of lights on and heating etc.”) to participation in local action groups (“I want to find out if there are any environmental action groups in my area and what I could do to help the environment where I live”).

In addition to the text initiating new behaviours, 25% of responses to this question mentioned that reading and engaging with the text reinforced or sustained existing pro-environmental efforts. For example, one respondent claimed that “It supported/accelerated what I'm already trying to do”, while another said “I was fairly engaged already but it motivated me to keep going and continue pushing for changes where I can influence things”. Elsewhere, one respondent alluded to a sense of strengthened hope amidst collective inaction: “By reading some of the examples, it was easy to see how small actions that individuals took turned into something significant within their communities. I often dismiss the thought that this is possible, so I found this challenging”. Finally, some respondents commented on the role of the text in increasing awareness and concern around inequality and injustice worldwide and links with environmental issues. For example, “I will be more aware of the impacts of my life choices on the environment and world at a wider scale.”

#### Attitudes and beliefs

Two questions enquired into impacts of the text on respondents’ attitudes and beliefs related to theology. They were (i) “Did ‘Saying Yes to Life’ change the way you think about God and creation? If so, how?”, and (ii) “Did ‘Saying Yes to Life’ change the way you think about Church, mission, or how Christians should live?”. A spread of responses was recorded to the first question regarding God and creation. Over 25% of responses indicated that the text did not change their thinking, with this being a combination of a few individuals who disagreed with the book’s messages and a larger number who already agreed. For example, one respondent reported “No [change], perhaps if it had been a more balanced book”, while another said “I already held the view that God did not give humans authority to exploit the environment”. Elsewhere there were suggestions that the book’s environmental messages represented a progressive political agenda, which was being uncritically adopted by the Church: “In places, it felt like this was agenda theology, with interpretation to fit an environmental message”. In contrast, 22% of respondents indicated some form of reinforcement, confirmation or further development of existing thinking, while 37% reported a change in thinking in some way. Many responses indicated elements of both, while responses from a small subset did not relate directly to the question posed. Comments that indicated a more profound or marked change related to decreased anthropocentrism and mastery-over-nature orientation (pro-dominion attitudes) (e.g. “I think there's still a residue of an old Pentecostal stance that we don't really look after the physical world … we focus only on the spiritual world … I definitely see that differently now!”). Others showed a decreased tendency toward dualistic perception of the physical world (nature/creation) as separate from and/or inferior to the spiritual world (e.g. “Yes. I am aware of God essentially in all living things, myself included in a way that I had not considered before”). Comments indicating reinforcement of beliefs spanned (i) increased awareness of God’s relationship to and concern for creation (“…so much more aware of how God and creation are intimately intertwined”), (ii) increased sense of obligation to care for creation (“I now feel a greater sense of responsibility to this planet, not just for future generations, but because it's something so close to God's heart”), (iii) increased appreciation for creation (“…really opened my eyes to other incredible habitats and creatures”), (iv) increased sense of participants’ own nature connectedness and/or interdependence (“I think it just enhanced my feeling that we are part of rather than over and above creation”), and (v) greater integration of beliefs about creation with soteriological (salvation) and eschatological (end of world) beliefs (“It really developed my thinking about the Creation story and all of Creation being part of redemption”).

Responses to the question “Did ‘Saying Yes to Life’ change the way you think about Church, mission, or how Christians should live?” revealed insights into the potential for collective action on the part of faith communities. As with the previous question, a number of responses emphasised a reinforcement of existing beliefs about the Church’s actions, e.g. “I don't think it changed the way I think about those things, but I think it has helped to re-inspire me to inspire my congregations to think about those things”. A second theme that emerged related to the inadequacy of the Church’s response to climate change and environmental crises. This was expressed in responses such as “the Church has failed badly to take appropriate action to advocate for the importance of climate care”, with some individuals suggesting that an inadequate response was impacting the Church’s modern-day relevance (e.g. “…if the church can grasp this opportunity, we may also follow the lead of young people, and rehabilitate their view of the church that for too long has been seen as irrelevant and out of touch”). A third theme emphasised the importance of integration of stewardship beliefs in church activities. For example, “I suppose I thought of it [environmental action] as slightly peripheral to the Church's main mission- the book emphasised how integral it actually is”. Finally, many responses mentioned an increase in perceived collective efficacy as part of wider Christian community. This included a sense that together Christians have a capacity to achieve substantial pro-environmental change (i.e. a practical benefit) as a function of being part of a likeminded, global faith community, and that being part of such a community helped respondents to feel less alone in their environmental concerns (i.e. an emotional benefit). The former point was articulated by one respondent as follows: “If the churches of the world were to mobilise towards effective environmental initiatives, our planetary problems could be solved”, with another expressing a similar sentiment: “The church could change the planet if it woke up to this reality and lead a global change”. The latter was exemplified by one participant as “How behind 'my' church is! Gosh, it is hard work sometimes so good to find I'm (we're) not alone”.

### Focus groups

The focus group discussions revealed experiences of participants in engaging with SYTL, processes of change in beliefs and behaviours, and relationships between internal and external systemic dimensions of sustainability. First, differences were evident in how intellectually accessible participants found the text to be. Many in Focus Group 1 were highly educated and familiar with academic writing and biblical theology, and demonstrated deep engagement with the book. In contrast, those less academically inclined (particularly Focus Group 2) found the text to be somewhat inaccessible. One participant, for example, explained that some of their Bible study members “found it very difficult. They said it was like reading a university document and couldn't cope with it”. Yet, for those in Focus Group 2, it was the inclusion of real-world stories, examples, and opportunities for discussion with others that enabled them to engage with the themes and respond. One participant, for example, explained “I also really loved the way that she [Valerio] drew on lots of other cultures and religious backgrounds as well… I think I find it a lot more personal, a lot more moving when you hear someone’s story”. Second, many focus group participants shared that it was engagement with associated spiritual practices that allowed the text’s environmental messages to have impact in their lives. The most prominent across all focus groups was appreciation of the transmutation of intellectual content into prayer. One participant explained that “Finishing with what we can pray about is good. [Prayer can] lead us into what we can actually do, and that's also an important feature that comes out of the book”. In addition to individual prayer, communal and congregational prayer was also mentioned, with one participant encouraged to integrate the environment into their regular Sunday morning service prayers.

Discussions about the processes through which SYTL influenced beliefs and behaviours revealed different responses, in accordance with results identified from the survey. The majority of participants, particularly in Focus Group 1 and Focus Group 3 experienced an affirmation of existing personal beliefs, rather than a change per se. Yet others did report a shift in beliefs. One participant recounted an experience of reflecting on their lifestyle: “I probably started reading it thinking that I was reasonably aware and responsible in terms of the way I act towards the planet, but then realised that I probably wasn't, not as much as I could be or should be”. Another expressed a deeper shift in beliefs: “it [the book] made me think very differently about how I want to look after things. It’s part of God and so of course I want to look after it”. In discussions about behaviour change, participants commonly connected their personal practices with theological reasoning, even though only 6 of the 15 participants reported making intentional alterations to their lifestyle. These individuals often expressed a greater sense of resolve to continue to try to live environmentally considerate lives. Interestingly, many participants referred to the use of stories and examples from around the world as helping to illustrate theological points. The principle of loving one’s neighbour was a particular focus, with one participant sharing that “being a Christian is nothing to do with loving your neighbour unless you look at the neighbour as part of the whole of God's creation”.

Finally, all focus groups commented on the need for change to go beyond the personal and spiritual domains to also encompass economic, political and social systems. One participant commented on how the text alerted him to ethical dimensions of global climate injustices: “I think that she's [Valerio] alerted me anew to the ethical dimensions of climate change and how particularly disadvantaged parts of the world are suffering a kind of double whammy: not only had they not had the benefits of industrialisation, but they’re suffering the consequences of it”. In accordance with this global perspective, participants highlighted the potential for a global faith community – churches, Christian charities and institutions, and other faith-based organisations – to influence environmental change on a wider economic, political and social scale, particularly when they adopt the beliefs, values, attitudes and behaviours described in the text.

These results suggest that there may be multiple pathways for effective engagement (not just cognitive) when designing interventions. The first pathway indicated primarily from Focus Groups 1 and 3 was a process of reading and personal reflection, which allowed a greater understanding of the links between theology, prayer and the environment, alongside new consideration of the role that faith can have in influencing economic, political and social systems. The second pathway was through discussion with others (mostly Focus Group 2). These participants were less “theologically-minded or deep-thinking” and were less environmentally aware before reading SYTL. Desired changes in attitudes and behaviours among these participants came through a process of discussing ideas, opinions and practices with others in the group.

## Discussion

### Synthesis of key findings

This study demonstrated that environmental messages couched explicitly within a religious tradition can bring about both external and internal change among people of faith. Externally, enhanced pro-environmental behavioural intentions were reported, with some behaviours shown to respond more readily to the intervention (e.g. recycling) than others (e.g. dietary choices). Additionally, qualitative evidence showed that these behavioural changes were recognised by participants as being expressions of internally held (religious) beliefs, values and worldviews and behaviour. This suggests that religious contexts may be especially important for activating latent beliefs and unleashing values (c.f. Chan et al. 2020) for environmental sustainability outcomes, especially among those whose existing beliefs are already conducive to environmental concern. Further, working within faith contexts to activate theological beliefs for sustainability outcomes may help to avoid the politicisation of environmental issues which can hamper broader system change. Quantitative results showed greater behaviour change among those with conservative political views, suggesting opportunities to relate to previously disengaged sectors of society who may otherwise dismiss environmental sustainability as a politically progressive agenda.

There was some evidence of changes in environmental attitudes and beliefs, connected to shifts in respondents’ worldview. Some of these shifts in belief touch on dimensions of what Hedlund-de Witt et al. (2014) operationalised as a “worldview”—including ontology (creation as sacred; rejection of Cartesian dualism; enhanced feeling of being “connected” to nature), anthropology (humanity’s role as stewards) and societal vision (the relevance of creation care).

We found evidence of a reduction in anthropocentrism among many participants. Past scholarship on religion and ecology has tended to compare beliefs, values and practices and teachings of major world religions to identify resources for environmental care (Jenkins et al. 2016). However, the present study demonstrates that it is possible to move towards bio/ecocentric perspectives *within* a particular religious framework, namely the Christian tradition, among particular individuals and communities. Most “greening of religion” studies have looked at broad longitudinal observations, yet this study’s pseudo-experimental design provides more practicable insights for designing effective interventions.

The integrated perspective on inner and outer change adopted in this study has received growing attention in sustainability science (Ives et al. 2020; Wamsler et al. 2021; Woiwode et al. 2021). Rather than beliefs, values and worldviews being considered as stable, linear predictors of pro-environmental behaviours, our study suggests a more closely entangled reality where internal and external change interact in complex ways (c.f. Maller 2021). In addition to shifts in worldview, for many participants, the intervention enabled an integration of beliefs—especially concern for nature with religious beliefs about salvation and end-times. Psychologists have long documented people’s ability to simultaneously hold inconsistent values and beliefs, often leading to cognitive or value-dissonance (Festinger 1957). This study suggests that such inconsistencies may similarly exist within religious contexts, and that carefully designed interventions may be helpful in aligning people’s environmental concerns with their faith convictions.

Connections between internal and external change were evident not only at individual scales; participants also referenced the potential for faith institutions to effect broader system change (e.g. “I would like to get more involved with inspiring societal, rather than just individual change”). Links between internal phenomena such as values, goals, mindsets and worldviews, and external structural or behavioural change have been recognised within conceptual frameworks for understanding sustainability transitions/transformation. Frameworks include leverage points (Abson et al. 2017), the “three spheres” of practical, personal and political transformation (O’Brien 2018), and discursive fields, or meanings related to “ideas, beliefs, expectations, knowledge, and other cognitive schemes” within socio-technical transitions (Pesch 2015). Yet religion’s role in enabling, constraining or interacting with system-level change has received scant research attention (Koehrsen 2015, 2018).

Survey free-text responses and focus group discussions revealed that various modes of engagement enabled behavioural and belief and worldview changes among different participants. Key practices included reading and personal reflection, dialogue and interaction, engaging with stories of people impacted by environmental degradation, and prayer. Focus group discussions revealed that some people have extensive existing knowledge (theological, scientific) and strong existing beliefs. They responded positively to logic and argument to integrate these beliefs and activate behaviour change or reinforcement. For others, it was a less intellectual exercise, with profound attitudinal and behavioural changes facilitated through interaction and discussion with others, as well as hearing stories. Both cases align with a social practice understanding of environmental behaviour change rather than simple, linear information deficit models (Hargreaves 2011). Yet our results additionally emphasise the under-explored potential of engaging spiritual practices (e.g. prayer and meditation) in initiating change for sustainability. This resonates with emerging research by some sustainability scholars, albeit outside explicitly religious contexts, such as mindfulness practices (Wamsler et al. 2017; Thiermann and Sheate 2021) and immersive educational experiences of indigenous cultures (Gray et al. 2021).

This study also suggests that even when presenting environmental messages in faith contexts, there is a need to attend to different preferences and personal dispositions of diverse audiences. While many individuals found the theology thought provoking and persuasive, others found it too intellectual. Still others found that practical recommendations were not realistic for many families’ financial position (“although I would love to shop organically with no packaging and only eating products grown or produced locally, I just can't afford to”). Audience segmentation research from secular environmental engagement initiatives may be just as relevant in faith contexts. Examples of this include the Britain Talks Climate project by Climate Outreach (Wang et al. 2020) and Global Warming’s Six Americas by the Yale Climate Communication Project (Leiserowitz et al. 2021).

In all focus groups, the importance of story-telling was highlighted as a way of reinforcing beliefs and inspiring a change in behaviour. Including stories from the Global South of the acute impacts of climate change and connecting these with the Christian teaching of loving one’s neighbour strengthened moral, personal and emotional connections across geographical divides. Storytelling is increasingly recognised as an important vehicle for environmental communication (Moezzi et al. 2017; Veland et al. 2018), and is likely to be important in faith contexts as well.

Additionally, dialogical engagement of subjects with the text’s content and other group participants pointed to the potential for broader narratives to be cultivated among faith communities. The role of dialogue and conversation in shaping beliefs and reinforcing commitments to act finds parallels in the notion of “discourse coalitions” (Riedy 2020): groups of actors who reproduce storylines and narratives. Given the importance of metanarratives in providing coherence and meaning to religious belief systems (Ives and Kidwell 2019), faith communities may have potential to unearth and reinforce “larger and thicker stories about human purpose, identity, duty, and responsibility” (Hulme 2020, p. 311) that could both mobilise and sustain action for sustainability. Yet, diverging views and opinions are common within faith communities, unlike the picture of homogenous sets of beliefs they are sometimes presented as containing. This was exemplified by one respondent’s comment: “As to how Christians should live, [discussions] revealed a huge difference of opinion amongst our group”. Nevertheless, this further emphasises the importance of spaces for reflexive dialogue where different views can be aired and processed, as is necessary for transformation-oriented learning for sustainability (Macintyre et al. 2018).

### Process of change and practical implications

**Fig. 1:**
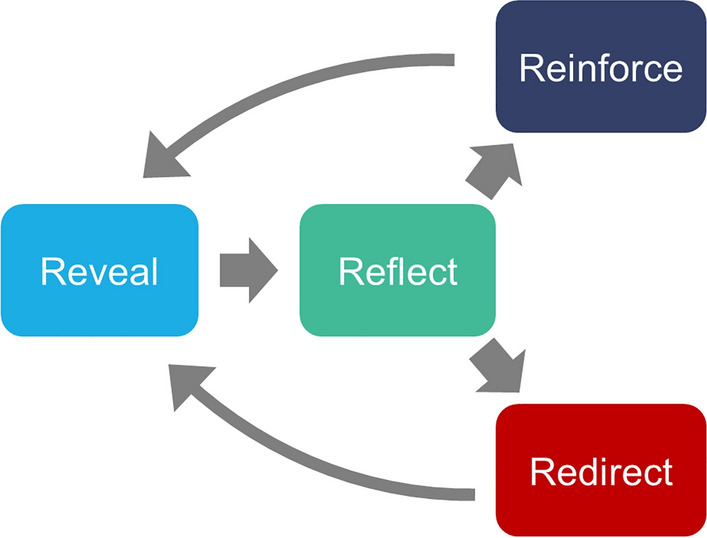
Schematic model of four stages of strengthening environmentalism among study participants. The reveal and reflect stages relate to engagement with and processing of new ideas in relation to existing beliefs and behaviours. The redirect stage concerns initiating new actions, while the reinforce stage is about integration of beliefs and sustaining behaviour. The model creates two simultaneous feedback loops which are able to bring about virtuous cycles of environmental stewardship among faith communities

Drawing on results from the survey and focus groups, we propose a model for faith-based engagement for environmental sustainability. There are four key stages involved: revealing, reflecting, redirecting and reinforcing (see Fig. 1). Revealing refers to illuminating the nature of environmental problems and presenting theological ideas related to the environment. One example of this stage from survey responses included “It made me realise how much guidance is in the Bible about our responsibilities for the world and nature”. Reflecting was the stage where subjects considered their own beliefs and lifestyles, and whether changes were required according to new spiritual or moral rationales. This was evident in the following survey comments: “I probably started reading it thinking that I was reasonably aware and responsible in terms of the way I act towards the planet, but then realized that I probably wasn't, not as much as I could be or should be” and “I now feel a greater sense of responsibility to this planet, not just for future generations, but because it's something so close to God's heart”. The third and fourth stages depended on whether individuals felt a need to change their behaviours or sustain existing behaviours. Redirecting was evident by commitments to shift actions and lifestyles, for example, “Going forward, I think I will put more thought into the products I buy and where they are sourced. I'll think more about whether it is made of recycled materials”. Reinforcing on the other hand was about strengthening commitments to act: “I was fairly engaged already but it motivated me to keep going and continue pushing for changes where I can influence things”. Yet reinforcing also contained a deeper dimension of integration of previously disparate beliefs and knowledge: “I thought my environmental views were seen as rather a side issue to the local church and my Christian faith but now I can see how very much they are together and integral parts of each other”.

These processes revealed a number of insights that are relevant to broader discourses about bringing change for sustainability. First, the need to *sustain* behaviour change may be one area where faith communities have an especially important role to play. While much attention has been directed to understanding how to change behaviour, system-wide transformation requires persistent, ongoing action, often in the face of opposition or resistance. It is plausible that latent beliefs do not merely require activating and “unleashing”, but continually re-activating and sustaining. Faith communities may be important settings for this reinforcement, especially when behaviour changes are inconvenient, costly or not yet socially normative. So, although in some cases, interventions such as this may be “preaching to the choir,” they may not otherwise continue to sing.

Relatedly, responses suggested a possible link between faith settings and perceived collective efficacy or “agentic influence” (Sachdeva 2016, p. 4). As such, there is an interesting intersection between religion and the psychological barriers to climate action of “perceived behavioural control and self-efficacy” presented by Gifford (2011). Rooted in Olson’s (1965) collective action problem, Gifford acknowledges individuals’ reluctance to act, assuming the negligible impact of measures taken relative to the magnitude of the climate crisis. Many study participants linked their collective religious identities with collective efficacy beliefs, trusting that as individual members of a like-minded global faith community, collectively their pro-environmental actions could effect substantial change. People of faith across many traditions are commonly encouraged to perceive of themselves as part of a globally interconnected community of believers, and this sense of unity has been identified as a source of agency for pro-environmental change. Indeed the Islamic notion of *Ummah* (the unity of Muslims) has been embraced as offering “an ethical order of trusteeship (Amana أمانة)” towards the climate (Youssef 2021), while the 2009 Hindu declaration on climate change emphasised that “as one sixth of the human family, Hindus can have a tremendous impact” (Convocation of Hindu Spiritual Leaders 2009). A sense of a global community of believers can bring geographical connection, as highlighted by one respondent: “I loved hearing about the response of Christians/churches in some of the countries most impacted by environmental issues, e.g. tree planting. It was inspirational and a challenge for us in the UK to be standing with our global brothers and sisters in protecting God’s world”. With research on social tipping points highlighting the need for a critical mass of committed individuals to initiate social change (Centola et al. 2018), faith communities may offer a context for building a sense of collective action, even across geographical and cultural divides (c.f. World Economic Forum 2016). The question of who initiates social transformation is receiving much attention in sustainability scholarship, with recent commentary highlighting the need for alignment of actors to initiate reinforcing cycles of ambition between governments and the private sector (Hsu et al. 2017). Yet with research emphasising the importance of civil society and social movements in initiating social change (Smith et al. 2020), our work suggests that faith settings may provide social infrastructure facilitating members’ collective action more easily than individuals lacking equivalent social structures.

### Future research

As an exploratory piece of research, results from this study have opened many new research avenues worthy of further investigation. Most obvious is the need to understand whether interventions in other religious and geographical contexts would elicit similar responses. Indeed, even in the UK, there is scope for further research among Christian communities given the dominance of Anglican participants in this study and the self-selection of respondents who are likely to have already been aligned with or sympathetic to the perspective of SYTL. Less environmentally engaged groups may exhibit greater cognitive and behavioural change in response, yet may also be more resistant to pro-environmental messaging. Indeed, even within the present sample, there was evidence of conflicting theological views—especially from more conservative positions—which would be worth exploring further.

There is also a need to further explore behaviour changed in response to the religiously framed environmental messaging. The introduction of COVID-19 government restrictions on movement in the UK at the time of the study also meant that the survey focussed on self-reported behavioural intentions. Thus, the effects observed here may not necessarily correspond to objective behaviour measures, although self-reported and actual behaviour are moderately correlated on average (Kormos and Gifford 2014). Therefore, we suggest further research on both actual documented behaviour change, as well as behaviours practiced over longer time periods, particularly as societies move towards a post-pandemic reality. This will help to understand how faith communities might help to sustain and normalise pro-environmental lifestyles, given the importance of social norms for sustainability (Nyborg et al. 2016). Additionally, explicitly attending to the unique contribution of faith contexts, and how scientific information interacts with deeply held belief systems, faith group dynamics and religious leadership structures would help understand more exactly the processes behind the results we observed.

Although quantitative measures of transcendental values did not show statistical responses to the intervention, the differences observed in the psychological constructs of the New Ecological Paradigm and Nature Relatedness along with qualitative insights into shifts in attitudes and beliefs suggest that further research into the formation and change of values, beliefs and worldviews in religious settings would be worthwhile. Value shift has been the topic of theoretical postulating and debate within social–ecological research (Ives et al. 2017; Manfredo et al. 2017; Kendal and Raymond 2019), with some recent empirical evidence suggesting population-level shifts towards biocentric values is observable (Manfredo et al. 2021). With calls to better understand the mechanisms for such changes, the role of religion in forming and shaping values at individual, community, and population scales is of great significance. Indeed, one pertinent question is whether environmental messaging *within* religious frameworks offers greater potential for pro-environmental cognitive shifts because the setting maintains sufficient familiarity and stability of belief systems such that people are open to being challenged to reflexively reconsider specific attitudes, beliefs and behaviours.

Given the emphasis of collective efficacy in qualitative survey responses, further research on how faith communities’ collective identity influences their perceived self- and collective efficacy would be worthwhile, along with how this translates to specific behaviours and particular theological views. Scholarship reveals a contested relationship between religious beliefs and what Rotter (1966) termed internal or external locus of control (LOC). Studies disagree as to whether religiosity, in particular, predicts inaction and passivity as a product of external LOC (Coursey et al. 2013). Nevertheless, belief in “suprahuman powers” is often associated with relinquishing personal agency and subsequent inertia (Gifford 2011). Indeed, a recent study of Christians in the U.S. revealed opposing influences of stewardship beliefs and belief in a controlling god with respect to pro-environmental support (Eom et al. 2021). Understanding which theological messages, including those concerned with identifying as part of a wider faith network, are most effectively activated for environmental outcomes would help both religious actors and policy-makers enhance positive engagement with faith communities.

Finally, there is a need to understand how religious institutions, communities and belief systems might help to mobilise necessary system transformations for sustainability. The role of faith communities as keystone social institutions in much of the world—especially the Global South—means that there is an urgent need to understand how faith-based interventions might activate the latent potential for societal transformation. Indeed, faith communities have historically had an instrumental role in transformative social justice movements such as abolitionism, and the anti-apartheid and civil rights movements (Smith 2014).

## Conclusion

This study has demonstrated the importance and largely untapped potential of working within religious belief systems to effect change for environmental sustainability. Evidence of internal shifts in beliefs and attitudes in response to pro-environmental messages framed through the lens of Christian theology, and the activation of these beliefs in self-reported behavioural changes offers substantial promise that faith communities can play an important role in the societal transformation needed for an ecologically sustainable twenty-first century. Further, this study has shown that a variety of intellectual, social, emotional and spiritual engagement pathways may be required to allow environmental messages to be integrated and enacted by individuals. The role of faith in sustaining pro-environmental behaviour and encouraging a sense of collective efficacy is likely to be especially important in this regard. We call for further empirical research on “the motivating power of the Sacred to environmental protectionism” (Sachdeva 2016, p. 1) and the particular opportunities afforded by faith contexts to mobilise environmental action.

## Supplementary Information

Below is the link to the electronic supplementary material.

Supplementary file1 (PDF 902 KB)

## Footnotes

1 “Mission” is defined by the Concise Oxford Dictionary of World Religions (2000) as “The sense of obligation in all religions to share their faith and practice with others”. The Church of England have adopted five “marks of mission”, namely (i) to proclaim the Good News of the Kingdom, (ii) to teach, baptise and nurture new believers, (iii) to respond to human need by loving service, (iv) to seek to transform unjust structures of society, to challenge violence of every kind and to pursue peace and reconciliation, and (v) to strive to safeguard the integrity of creation and sustain and renew the life of the earth.
