# Supplementary material for: Activating faith: pro-environmental responses to a Christian text on sustainability
Source: Sustain Sci. 2022 Aug 22;18(2):877–90. doi: 10.1007/s11625-022-01197-w (PMC9395780; doi:10.1007/s11625-022-01197-w)
Supplement: Supplementary file 1 — Supplementary file1 (PDF 902 KB) [file 11625_2022_1197_MOESM1_ESM.pdf]

Introduction

.

Research Project: "Saying Yes to Life"

Thank you for your interest in this survey. Your views will help us understand how people's thoughts about environmental and social issues are shaped by the Archbishop of Canterbury's Lent book "Saying Yes to Life" by Ruth Valerio. Findings will be shared with Lambeth Palace, SPCK and Tearfund.

The research

This survey is designed to be completed before or in the early stages of interacting with "Saying Yes to Life". You are invited to contribute to this study by responding to some questions about your thoughts and opinions about the environment and sustainability. We are looking for people who are engaging with the book "Saying Yes to Life" in some way: perhaps by reading it, discussing it in a small group, or using the associated online resources. The information you provide will help to understand how your views might change over time, and will aid in the development of future resources on this topic. This questionnaire should take about 10 minutes to complete.

Who is running the project?

The project is led by Dr. Christopher Ives (School of Geography, University of Nottingham). The project will adhere to the University of Nottingham's [Code of Research Conduct and Research Ethics].

Do I have to take part?

Your participation is entirely voluntary, and even if you decide to begin the survey you are still free to stop at any time and without giving a reason. You are free to request that any information you have provided be destroyed, even if you have completed only part of the survey.

What will happen to the information I provide?

The survey is completed anonymously and any written responses used in written and published output will not be personally identifiable. Any personal information (e.g. e-mail addresses) will be used only by the research team and not distributed further. Information will be stored securely on password protected servers hosted by the University of Nottingham. All treatment and storage of data will adhere to the University of Nottingham's Data Protection Policy and Guidelines.

How will the research be used?

Summarised information gathered from the research will contribute to the writing of academic journal articles and will be shared with Tearfund. Results may also be presented at academic conferences and other University settings.

Are there any risks to me participating?

It is not anticipated that completing this survey will expose you to any personal risks, however some of the questions ask you about your beliefs, values in life and your lifestyle. If you would rather not answer these questions you may leave the survey at any time.

Want to know more?

If you have any questions or comments, please email me at chris.ives@nottingham.ac.uk

Thank you for your help!

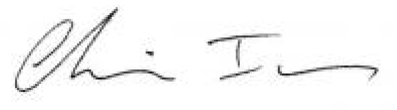

Dr. Chris Ives

Assistant Professor

School of Geography

University of Nottingham

University Park

Nottingham, NG7 2RD

E: chris.ives@nottingham.ac.uk

T: +44(0)115 8232536

. I confirm that I am over 18, have read the above information for participants, and agree to take part in this survey.

Yes

No

Introduction

Q1. How did you first hear about the book "Saying Yes to Life"?

Media (e.g. newspaper, television)

Social media (e.g. Twitter, Facebook, Instagram)

A friend

My church or faith community

Bookshop catalogue

Other (please specify below)

Q2. How do you intend to engage with "Saying Yes to Life" (check all that apply)

Reading the book on my own

Reading and discussing the book with others (e.g. home group, church, Bible study, book club)

Following studies or resources based on the book, but not reading the book itself.

Using it to aid communal worship or liturgy

Using it to aid personal prayer, meditation or other personal spiritual practice

Other (please specify below)

Questions on what you think about the environment in general

Q3.

Listed below are statements about the relationship between humans and the environment. For each one, please indicate whether you STRONGLY AGREE, MILDLY AGREE, are UNSURE, MILDLY DISAGREE or STRONGLY DISAGREE with it.

	Strongly agree	Mildly agree	Unsure	Mildly disagree	Strongly disagree
If things continue on their present course, we will soon experience a major ecological catastrophe	○	○	○	○	○
Humans are severely abusing the environment	○	○	○	○	○
Humans have the right to modify the natural environment to suit their needs	○	○	○	○	○

	Strongly agree	Mildly agree	Unsure	Mildly disagree	Strongly disagree
When humans interfere with nature it often produces disastrous consequences	○	○	○	○	○
The earth is like a spaceship with very little room and resources	○	○	○	○	○
	Strongly agree	Mildly agree	Unsure	Mildly disagree	Strongly disagree
The so-called "ecological crisis" facing humankind has been greatly exaggerated	○	○	○	○	○
Humans will eventually learn enough about how nature works to be able to control it	○	○	○	○	○
The balance of nature is very delicate and easily upset	○	○	○	○	○
Humans were meant to rule over the rest of nature	○	○	○	○	○
The balance of nature is strong enough to cope with the impacts of modern industrial nations	○	○	○	○	○
	Strongly agree	Mildly agree	Unsure	Mildly disagree	Strongly disagree
Despite our special abilities humans are still subject to the laws of nature	○	○	○	○	○
Plants and animals have as much right as humans to exist	○	○	○	○	○
We are approaching the limit of the number of people the earth can support	○	○	○	○	○
Human ingenuity will ensure that we do NOT make the earth unlivable	○	○	○	○	○
The earth has plenty of natural resources if we can just learn to develop them	○	○	○	○	○

Questions about your lifestyle

Q4. Below are some personal actions that relate to the environment. How often do you do the following?

	Always	Very often	Quite often	Not very often	Never	Can't do this/not applicable
Use public transport (e.g. bus, train) rather than travel by car (all journeys)	○	○	○	○	○	○
Compost your kitchen waste	○	○	○	○	○	○
Take part in a protest about an environmental issue	○	○	○	○	○	○
Walk or cycle for short journeys less than 2 or 3 miles	○	○	○	○	○	○
Avoid eating meat	○	○	○	○	○	○
Write to your MP when concerned about an environmental issue	○	○	○	○	○	○
Car share with others who need to make a similar journey	○	○	○	○	○	○

	Always	Very often	Quite often	Not very often	Never	Can't do this/not applicable
Volunteer for an environmental cause	○	○	○	○	○	○
Put more clothes on when you feel cold rather than putting the heating on or turning it up	○	○	○	○	○	○
Take your own shopping bag when shopping	○	○	○	○	○	○
Take fewer flights when possible	○	○	○	○	○	○
Switch off lights in rooms that aren't being used	○	○	○	○	○	○
Buy recycled paper products such as toilet paper or tissues	○	○	○	○	○	○
Reuse or repair items instead of throwing them away	○	○	○	○	○	○

	Always	Very often	Quite often	Not very often	Never	Can't do this/not applicable
Eat food which is organic, locally-grown or in season	○	○	○	○	○	○
Keep the tap running while you brush your teeth	○	○	○	○	○	○

	Always	Very often	Quite often	Not very often	Never	Can't do this/not applicable
Leave your TV on standby for the night	○	○	○	○	○	○
Pray for God's creation	○	○	○	○	○	○
Decide not to buy something because you feel it has too much packaging	○	○	○	○	○	○

Q5. Now please respond to the following questions about your home energy.

	Yes, we already buy	Yes, we are seriously considering	No	Considered in past and rejected
Have you installed or are you seriously considering installing solar water heating?	○	○	○	○
Does your household buy or is seriously considering buying its electricity on a Green Tariff?	○	○	○	○
Have you installed or are you seriously considering installing solar panels for electricity?	○	○	○	○
Have you installed or are you seriously considering installing wind turbine to generate electricity?	○	○	○	○

Q6. What is your mode of transport to work (or the destination you travel most regularly to)?

Private transport, including getting lifts	Public transport	Active travel	Work from home

Q7. How often do you separate items for recycling?

Always	Usually	Sometimes	Never	No recycling facilities, use normal rubbish collection

Q8. How frequently do you use the following recycling facilities?

	Very often	Fairly often	Not very often	Never	No recycling facility in area
A paper recycling point	○	○	○	○	○
A bottle bank	○	○	○	○	○
A garden waste recycling facility	○	○	○	○	○
A plastic bag recycling point	○	○	○	○	○

Questions about what is important to you in life

Q9. Below are some statements about general life values. Please indicate the importance of each of the following as a GUIDING PRINCIPLE IN YOUR LIFE.

Wealth (material possessions, money)	⌄
Equality (equal opportunity for all)	⌄
Protecting the environment (preserving nature)	⌄
Preventing pollution (protecting natural resources)	⌄
Social justice (correcting injustice, care for the weak)	⌄
Helpful (working for the welfare of others)	⌄
A world at peace (free of war and conflict)	⌄
Authority (the right to lead or command)	⌄
Unity with nature (fitting into nature)	⌄
Social power (control over others, dominance)	⌄
Influential (having an impact on people and events)	⌄
Respecting the earth (harmony with other species)	⌄

Questions about your connection with nature.

Q10. The following questions are about how you think about nature. For each of the following, please rate the extent to which you agree with each statement. Please respond as you really feel, rather than how you think "most people" feel.

	Disagree strongly	Disagree a little	Neither agree nor disagree	Agree a little	Agree strongly

	Disagree strongly	Disagree a little	Neither agree nor disagree	Agree a little	Agree strongly
My connection to nature and the environment is part of my spirituality.	○	○	○	○	○
My ideal vacation spot would be a remote, wilderness area.	○	○	○	○	○
I take notice of wildlife wherever I am.	○	○	○	○	○
I always think about how my actions affect the environment.	○	○	○	○	○
I feel very connected to all living things and the earth.	○	○	○	○	○
My relationship to nature is an important part of who I am.	○	○	○	○	○

Questions about your faith

Q11. How important is faith or religion in your life?

Extremely important

Very important

Moderately important

Slightly important

Not at all important

Q12. How regularly do you participate in corporate religious activity (e.g. attending church, Mass, Bible study)

Daily

2-3 times a week

Once a week

Fortnightly

Monthly

Less than once a month

Q13. How regularly do you engage in personal religious or spiritual practices (e.g. prayer, meditation, contemplation, fasting)

Multiple times per day

Daily

2-3 times a week

Once a week

Fortnightly

Monthly

Less than once a month

Q14. Please indicate how much you agree with the following statements related to the Bible.

	Disagree strongly	Disagree a little	Unsure	Agree a little	Agree strongly
The Bible is the actual word of God and we should read it and apply it literally.	○	○	○	○	○
The Bible is the inspired word of God, and was written by human beings from particular social and cultural perspectives, and therefore needs interpretation to apply it today.	○	○	○	○	○
The Bible is an ancient book of history and legends; God had nothing to do with it.	○	○	○	○	○

Q15. The following statements describe reasons why we might care for the environment. Please indicate how much you agree or disagree with the following:

	Disagree strongly	Disagree a little	Unsure	Agree a little	Agree strongly
Our current actions to care for the earth matter because we participate in God's restoration of creation in ways that endure into the New Creation.	○	○	○	○	○

	Disagree strongly	Disagree a little	Unsure	Agree a little	Agree strongly
Our current actions to care for the earth matter because they reflect what God will one day do in a new creation, even if the effects of our actions don't last.	○	○	○	○	○
Our current actions to care for the earth do not matter because God will one day discard this creation.	○	○	○	○	○

Q16. Which Christian denomination do you identify with?

Anglican
Baptist
Catholic
Episcopalian
Methodist
Orthodox
Presbyterian
Pentecostal
None
Other

Questions about you

Q17. What is your gender?

Male
Female
Other
Prefer not to say

Q18. What is your age?

18 - 24
25 - 34
35 - 44
45 - 54
55 - 64
65 - 74
75 - 84

85 or older

Prefer not to say

Q19. What is the highest level of education you have completed?

Less than high school

High school graduate

Diploma or professional qualification

Undergraduate university degree

Masters university degree

Doctorate

Q20. What is your household type?

Single, no children

Single, children 0-17

Couple/adults, no children

Couple/adults, children 0-17

Q21. On a rating between 1 and 9, how would you perceive your economic status?

1 (Lower income)

2

3

4

5

6

7

8

9 (Upper income)

Prefer not to say

Q23. In general, how would you describe your political views?

Extremely liberal

Very liberal

Somewhat liberal

Centre

Somewhat conservative

Very conservative

Extremely conservative

Prefer not to say

Q24. What country do you live in?

Q25. If you live in the UK, what is the first half of your postcode? (e.g. SW1)

Q26. What is your household residential location?

Urban

Small town

Village, rural

Finishing up

Q28. We are interested in your experience of engaging with "Saying Yes to Life". As such, it would be very helpful to ask you a few more questions after you've completed the book. **Would you be happy to be sent a follow-up survey?**

Yes

No

Q28a. Please provide an email address for us to send you a follow-up survey.

Q29. If you are working through the book as part of a church or smaller study group and would be happy for us to contact you about your experience, please list the name of your church and any relevant contact details below.

Q27. If you have any additional comments, please share them below.

Powered by Qualtrics

Introduction

.

Research Project: "Saying Yes to Life"

Thank you for your interest in this survey. Your views will help us understand how people's thoughts about environmental and social issues are shaped by the Archbishop of Canterbury's Lent book "Saying Yes to Life" by Ruth Valerio. Findings will be shared with Lambeth Palace, SPCK and Tearfund.

The research

This survey is the second of two surveys designed to understand people's thoughts and experienced related to engaging with "Saying Yes to Life". This particular survey should be completed ***after*** you have finished reading the book or working though related materials. The information you provide will help to understand how your views may have changed over time, and will aid in the development of future resources on this topic. This questionnaire should take about 15 minutes to complete.

If you have completed the preliminary survey, you may notice that you are asked a number of the same questions again. While this may seem strange, it is important that you do complete these questions as your answers will help us to understand the degree to which "Saying Yes to Life" has, or has not, shaped your views.

Who is running the project?

The project is led by Dr. Christopher Ives (School of Geography, University of Nottingham). The project will adhere to the University of Nottingham's Code of Research Conduct and Research Ethics.

Do I have to take part?

Your participation is entirely voluntary, and even if you decide to begin the survey you are still free to stop at any time and without giving a reason. You are free to request that any information you have provided be destroyed, even if you

have completed only part of the survey.

What will happen to the information I provide?

The survey is completed anonymously and any written responses used in written and published output will not be personally identifiable. Any personal information (e.g. e-mail addresses) will be used only by the research team and not distributed further. Information will be stored securely on password protected servers hosted by the University of Nottingham. All treatment and storage of data will adhere to the University of Nottingham's Data Protection Policy and Guidelines.

How will the research be used?

Summarised information gathered from the research will contribute to the writing of academic journal articles and will be shared with Tearfund. Results may also be presented at academic conferences and other University settings.

Are there any risks to me participating?

It is not anticipated that completing this survey will expose you to any personal risks, however some of the questions ask you about your beliefs, values in life and your lifestyle. If you would rather not answer these questions you may leave the survey at any time.

Want to know more?

If you have any questions or comments, please email me at chris.ives@nottingham.ac.uk

Thank you for your help!

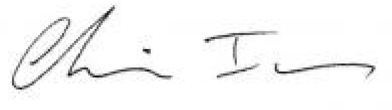

Dr. Chris Ives
Assistant Professor
School of Geography
University of Nottingham
University Park
Nottingham, NG7 2RD
E: chris.ives@nottingham.ac.uk
T: +44(0)115 8232536

. I confirm that I am over 18, have read the above information for participants, and agree to take part in this survey.

Yes

No

Participation in previous survey

i. Did you complete the initial survey about “Saying Yes to Life” before or in the early stages of reading the book?

Yes

No

ii. Please enter the unique survey code you were emailed. (If you **do not** have access to the code but did complete the previous survey, please enter your e-mail address instead).

Introduction

Q1. How did you first hear about the book "Saying Yes to Life"?

Media (e.g. newspaper, television)

Social media (e.g. Twitter, Facebook, Instagram)

A friend

My church or faith community

Bookshop catalogue

Other (please specify below)

Q2. How did you engage with "Saying Yes to Life" (check all that apply)

Reading the book on my own

Reading and discussing the book with others (e.g. home group, church, Bible study, book club)

Following studies or resources based on the book, but not reading the book itself.

Using it to aid communal worship or liturgy

Using it to aid personal prayer, meditation or other personal spiritual practice

Other (please specify below)

Q3. Did Covid-19 and its associated impacts influence your experience of working through "Saying Yes to Life"? If so, how?

Questions on what you think about the environment in general

Q4.

Listed below are statements about the relationship between humans and the environment. For each one, please indicate whether you STRONGLY AGREE, MILDLY AGREE, are UNSURE, MILDLY DISAGREE or STRONGLY DISAGREE with it.

	Strongly agree	Mildly agree	Unsure	Mildly disagree	Strongly disagree
The balance of nature is strong enough to cope with the impacts of modern industrial nations	○	○	○	○	○
If things continue on their present course, we will soon experience a major ecological catastrophe	○	○	○	○	○
We are approaching the limit of the number of people the earth can support	○	○	○	○	○
Plants and animals have as much right as humans to exist	○	○	○	○	○
The earth has plenty of natural resources if we can just learn to develop them	○	○	○	○	○

	Strongly agree	Mildly agree	Unsure	Mildly disagree	Strongly disagree
Humans are severely abusing the environment	○	○	○	○	○
Humans were meant to rule over the rest of nature	○	○	○	○	○
The earth is like a spaceship with very little room and resources	○	○	○	○	○
Human ingenuity will ensure that we do NOT make the earth unlivable	○	○	○	○	○

	Strongly agree	Mildly agree	Unsure	Mildly disagree	Strongly disagree
Humans will eventually learn enough about how nature works to be able to control it	○	○	○	○	○

	Strongly agree	Mildly agree	Unsure	Mildly disagree	Strongly disagree
When humans interfere with nature it often produces disastrous consequences	○	○	○	○	○
The balance of nature is very delicate and easily upset	○	○	○	○	○
Humans have the right to modify the natural environment to suit their needs	○	○	○	○	○
The so-called "ecological crisis" facing humankind has been greatly exaggerated	○	○	○	○	○
Despite our special abilities humans are still subject to the laws of nature	○	○	○	○	○

Questions about your lifestyle

Q5. Below are some personal actions that relate to the environment. After engaging with "Saying Yes to Life", how often do you perform, or intend to perform the following actions?

	Always	Very often	Quite often	Not very often	Never	Can't do this/not applicable
Car share with others who need to make a similar journey	○	○	○	○	○	○
Take your own shopping bag when shopping	○	○	○	○	○	○
Pray for God's creation	○	○	○	○	○	○
Reuse or repair items instead of throwing them away	○	○	○	○	○	○
Volunteer for an environmental cause	○	○	○	○	○	○
Take part in a protest about an environmental issue	○	○	○	○	○	○

	Always	Very often	Quite often	Not very often	Never	Can't do this/not applicable
Use public transport (e.g. bus, train) rather than travel by car (all journeys)	○	○	○	○	○	○

	Always	Very often	Quite often	Not very often	Never	Can't do this/not applicable
Write to your MP when concerned about an environmental issue	○	○	○	○	○	○
Decide not to buy something because you feel it has too much packaging	○	○	○	○	○	○
Switch off lights in rooms that aren't being used	○	○	○	○	○	○
Avoid eating meat	○	○	○	○	○	○
Leave your TV on standby for the night	○	○	○	○	○	○
Compost your kitchen waste	○	○	○	○	○	○
Walk or cycle for short journeys less than 2 or 3 miles	○	○	○	○	○	○

	Always	Very often	Quite often	Not very often	Never	Can't do this/not applicable
Keep the tap running while you brush your teeth	○	○	○	○	○	○
Eat food which is organic, locally-grown or in season	○	○	○	○	○	○
Buy recycled paper products such as toilet paper or tissues	○	○	○	○	○	○
Put more clothes on when you feel cold rather than putting the heating on or turning it up	○	○	○	○	○	○
Take fewer flights when possible	○	○	○	○	○	○

Q6. Now please respond to the following questions about your home energy.

	Yes, we already buy	Yes, we are seriously considering	No	Considered in past and rejected	Not Applicable
Have you installed or are you seriously considering installing solar panels for electricity?	○	○	○	○	○
Have you installed or are you seriously considering installing wind turbine to generate electricity?	○	○	○	○	○
Does your household buy or is seriously considering buying its electricity on a Green Tariff?	○	○	○	○	○
Have you installed or are you seriously considering installing solar water heating?	○	○	○	○	○

Q7. What is your actual, or intended, mode of transport to work (or the destination you travel most regularly to)?

Private transport, including getting lifts	Public transport	Active travel	Work from home	Not Applicable

Q8. How often do you separate items for recycling?

Always	Usually	Sometimes	Never	No recycling facilities, use normal rubbish collection	Not Applicable

Q9. How frequently do you use, or intend to use, the following recycling facilities?

	Very often	Fairly often	Not very often	Never	No recycling facility in area	Not Applicable
A garden waste recycling facility	○	○	○	○	○	○
A bottle bank	○	○	○	○	○	○
A paper recycling point	○	○	○	○	○	○
A plastic bag recycling point	○	○	○	○	○	○

Questions about what is important to you in life

Q10. Below are some statements about general life values. Please indicate the importance of each of the following as a GUIDING PRINCIPLE IN YOUR LIFE.

Social power (control over others, dominance)	
A world at peace (free of war and conflict)	
Social justice (correcting injustice, care for the weak)	
Preventing pollution (protecting natural resources)	
Unity with nature (fitting into nature)	
Influential (having an impact on people and events)	
Authority (the right to lead or command)	
Equality (equal opportunity for all)	
Respecting the earth (harmony with other species)	
Protecting the environment (preserving nature)	
Wealth (material possessions, money)	
Helpful (working for the welfare of others)	

Questions about your connection with nature.

Q11. The following questions are about how you think about nature. For each of the following, please rate the extent to which you agree with each statement. Please respond as you really feel, rather than how you think "most people" feel.

	Disagree strongly	Disagree a little	Neither agree nor disagree	Agree a little	Agree strongly
I always think about how my actions affect the environment.	○	○	○	○	○
My ideal vacation spot would be a remote, wilderness area.	○	○	○	○	○
My connection to nature and the environment is part of my spirituality.	○	○	○	○	○
I feel very connected to all living things and the earth.	○	○	○	○	○

	Disagree strongly	Disagree a little	Neither agree nor disagree	Agree a little	Agree strongly
My relationship to nature is an important part of who I am.	O	O	O	O	O
I take notice of wildlife wherever I am.	O	O	O	O	O

Your thoughts about "Saying Yes to Life"

. Below are some questions about your thoughts and experiences of working through "Saying Yes to Life". Please write as much or as little as you feel comfortable with.

Q12. What did you find most challenging, intriguing or interesting from working through the book and/or online resources? This could be, for example, a specific chapter or an idea that grabbed your attention.

Q13. Did you disagree with anything? If so, what?

Q14. Did "Saying Yes to Life" change the way you intend to live your life? If so, how?

Q15. Did "Saying Yes to Life" change the way you think about God and creation? If so, how?

Q16. Did "Saying Yes to Life" change the way you think about the Church, mission, or how Christians should live? If so, how?

Q17. Covid-19 is an extraordinary crisis that has had a profound human cost, stretched health systems, and impacted economies and livelihoods. In the months to come, there will be an opportunity for nations and communities to rethink and rebuild society. After reading “Saying Yes to Life” do you have any insights about what society should value or how it should look in the future?

Questions about your faith

Q18. How important is faith or religion in your life?

Extremely important
Very important
Moderately important
Slightly important
Not at all important

Q19. How regularly do you participate in corporate religious activity (e.g. attending church, Mass, Bible study)

Daily
2-3 times a week
Once a week
Fortnightly
Monthly
Less than once a month

Q20. How regularly do you engage in personal religious or spiritual practices (e.g. prayer, meditation, contemplation, fasting)

Multiple times per day

Daily

2-3 times a week

Once a week

Fortnightly

Monthly

Less than once a month

Q21. Please indicate how much you agree with the following statements related to the Bible.

	Disagree strongly	Disagree a little	Unsure	Agree a little	Agree strongly
The Bible is the actual word of God and we should read it and apply it literally.	○	○	○	○	○
The Bible is the inspired word of God, and was written by human beings from particular social and cultural perspectives, and therefore needs interpretation to apply it today.	○	○	○	○	○
The Bible is an ancient book of history and legends; God had nothing to do with it.	○	○	○	○	○

Q22. The following statements describe reasons why we might care for the environment. Please indicate how much you agree or disagree with the following:

	Disagree strongly	Disagree a little	Unsure	Agree a little	Agree strongly
Our current actions to care for the earth matter because we participate in God's restoration of creation in ways that endure into the New Creation.	○	○	○	○	○

	Disagree strongly	Disagree a little	Unsure	Agree a little	Agree strongly
Our current actions to care for the earth matter because they reflect what God will one day do in a new creation, even if the effects of our actions don't last.	○	○	○	○	○
Our current actions to care for the earth do not matter because God will one day discard this creation.	○	○	○	○	○

Q23. Which Christian denomination do you identify with?

Anglican

Baptist

Catholic

Episcopalian

Methodist

Orthodox

Presbyterian

Pentecostal

Click to write Choice 11

None

Other

Questions about you

Q24. What is your gender?

Male

Female

Other

Prefer not to say

Q25. What is your age?

18 - 24

25 - 34

35 - 44

45 - 54

55 - 64

65 - 74

75 - 84

85 or older

Prefer not to say

Q26. What is the highest level of education you have completed?

Less than high school

High school graduate

Diploma or professional qualification

Undergraduate university degree

Masters university degree

Doctorate

Q27. What is your household type?

Single, no children

Single, children 0-17

Couple/adults, no children

Couple/adults, children 0-17

__________ Other

Q28. On a rating between 1 and 9, how would you perceive your economic status?

1 (Lower income)

2

3

4

5

6

7

8

9 (Upper income)

Prefer not to say

Q29. In general, how would you describe your political views?

Extremely liberal

Very liberal

Somewhat liberal

Centre

Somewhat conservative

Very conservative

Extremely conservative

Prefer not to say

Q30. What country do you live in?

Q31. If you live in the UK, what is the first half of your postcode? (e.g. SW1)

Q32. What is your household residential location?

Urban

Small town

Village, rural

Finishing up

Q33. If you are interested in contributing further to this research via a phone or web-based interview, please provide your email address below.

Q34. If you have any additional comments, please share them below.

Powered by Qualtrics
